# Composite SCC*mec* Element in Single-locus Variant (ST217) of Epidemic MRSA-15 Clone

**DOI:** 10.3201/eid2005.130934

**Published:** 2014-05

**Authors:** Carla Vignaroli, Alessio Mancini, Pietro E. Varaldo

**Affiliations:** Università Politecnica delle Marche, Ancona, Italy

**Keywords:** epidemic methicillin-resistant *Staphylococcus aureus*, MRSA, bacteria, antibacterial drugs, antibiotics, oxacillin, ciprofloxacin, resistance

**To the Editor:** Since early epidemiologic studies of methicillin-resistant *Staphylococcus aureus* (MRSA) were published, it has been clear that the majority of nosocomial MRSA infections worldwide are caused by isolates derived from a few highly epidemic MRSA (EMRSA) clones. These are thought to have emerged through acquisition of the staphylococcal cassette chromosome *mec* (SCC*mec*) element by successful methicillin-susceptible *S. aureus* strains, within 5 major lineages or clonal complexes (CCs) including CC22 (*1*). Although epidemic clones are found worldwide, shifts of the predominant clones over time in which the emerging and usually more antibacterial drug–susceptible clones replace the older ones have been noted in countries, in small regions within countries, and in single hospitals (*2*). The reasons and mechanisms of such replacement as well as the epidemiologic dynamics leading to the success of a particular epidemic clone are largely unknown.

In Italy, isolations of classical EMRSA clones such as ST8-MRSA-I, ST247-MRSA-I, and ST239-MRSA-III decreased from the 1990s to the 2000s; during the same period ST228-MRSA-I increased, became established, and turned into the predominant clone in Italy (*3*). The genesis of other clones, such as ST8-MRSA-IV and ST22-MRSA-IV, which were associated with a tendency towards decreased multidrug resistance, was documented during 2000–2007 (*3*). Similar to occurrences in other European countries, the gentamicin-susceptible Panton-Valentine leukocidin–negative ST22-MRSA-IV clone, also known as EMRSA-15 (*1*), is now becoming predominant in Italy, replacing ST228-MRSA-I in hospital settings (*4*).

As part of another investigation, we recently isolated a MRSA strain from the nasal swab samples of a 5-year-old boy and his parents. The 3 isolates shared the same antibacterial drug resistance pattern (oxacillin and ciprofloxacin resistance) and proved to be identical by pulsed-field gel electrophoresis, SCC*mec* typing, and *agr* typing. Remarkably, ≈2 months earlier, the child had been admitted to a pediatric hospital for 10 days to be evaluated and treated for behavioral problems. A MRSA isolate, which was identified in a nasal sample obtained and analyzed just before discharge in the absence of clinical symptoms and was not further investigated, showed the same antibacterial drug resistance pattern as the 3 isolates collected later. In the absence of an epidemiologic history of exposure outside the hospital, it seems reasonable to assume that the strain was acquired by the child in the hospital and then transmitted to his parents.

Investigation of the genetic background of the strain isolated from the child’s specimen, designated as Lu1, led to its assignment as ST217, a single-locus variant of EMRSA-15 within the same CC, CC22 (http://saureus.mlst.net), *agr* group I, and *spa* type t965. At times associated with ST22-MRSA-IV strains, t965 is a single repeat variant of t032, which is the most prevalent *spa* type of EMRSA-15; t965 and closely related *spa* types have been reported mainly in Germany and the United Kingdom (http://spa.ridom.de). Strain Lu1 was Panton-Valentine leukocidin–negative and lacked the ACME (arginine catabolic mobile element) cluster. By using current criteria (*5*), the SCC*mec* element was assigned to type IV(2B), which is consistent with the combination of a class B *mec* complex and a *ccrA2B2* (type 2) *ccr* complex. However, an additional *ccr* locus (*ccrC*, type 5) was found in the J3 region between *orfX* and IS*431*. The SCC*mec* element was thus identified as a composite type IV(2B&5). Sequencing of the *ccrC*-IS*431* segment (3,217 bp, GenBank accession no. HG315670), performing analysis by using BLAST (http://blast.ncbi.nlm.nih.gov), displayed variable alignment scores and high-level nucleotide identities to the corresponding regions found downstream of *ccrC* in the SCC*mec* elements of MRSA reference strains of types III(3A), IV(2B&5), V(5C2&5), and VII(5C1) (*5*). The highest identity (3,216/3,217 nt) was with JCSC6944 (GenBank accession no. AB505629), an unspecified animal isolate of type V(5C2&5) from Japan, subtype c, belonging to the livestock-associated MRSA clone CC398 (*6*).

Very few ST217 strains, and none from Italy, are currently found in the MLST database (http://saureus.mlst.net), and data on such strains are scant in the literature. In particular, ST217-MRSA-IV was 1 of the dominant MRSA lineages isolated from patients in a hospital in Switzerland (*7*) and was detected in food samples of animal origin in Spain (*8*).

The composite SCC*mec* organization we detected in strain Lu1, featuring 2 *ccr* complexes (type 2 and 5), is similar to that described in 2 isolates that belong to different genetic lineages: 1 (ST100, CC5), later designated ZH47 (*5*,*9*), was identified in a sample from an inpatient in Switzerland (*7*); and the other (ST59, CC59, community-associated MRSA) from a pediatric patient in Taiwan (*10*).

Strain Lu1 (ST217, a single-locus variant of EMRSA-15) might have evolved from the ST22-MRSA-IV clone, which has recently been identified in hospitals in Italy (*3*,*4*). Its SCC*mec* organization may result from recombination events in which a type IV(2B) element acquired the *ccrC*-containing region downstream of *orfX* from SCC*mec* elements that normally contain it (*5*). However, a genetic exchange involving MRSA strains of animal origin cannot be excluded, considering the virtually identical sequence of the *ccrC*-IS*431* segment shared by strains Lu1 and JCSC6944, the latter being a CC398 LA-MRSA (*6*), and the isolation of ST217 strains from food samples of animal origin (*8*).
